# Human mitochondrial ADP/ATP carrier SLC25A4 operates with a ping‐pong kinetic mechanism

**DOI:** 10.15252/embr.202357127

**Published:** 2023-06-06

**Authors:** Camila Cimadamore‐Werthein, Stephany Jaiquel Baron, Martin S King, Roger Springett, Edmund RS Kunji

**Affiliations:** ^1^ Medical Research Council Mitochondrial Biology Unit The Keith Peters Building, Cambridge Biomedical Campus Cambridge UK; ^2^ CellSpex, Kettering Northamptonshire UK

**Keywords:** adenine nucleotide translocator, ADP/ATP translocase, bioenergetics, mitochondrial carrier family, SLC25, Membranes & Trafficking, Metabolism, Organelles

## Abstract

The mitochondrial ADP/ATP carrier (SLC25A4), also called the adenine nucleotide translocase, imports ADP into the mitochondrial matrix and exports ATP, which are key steps in oxidative phosphorylation. Historically, the carrier was thought to form a homodimer and to operate by a sequential kinetic mechanism, which involves the formation of a ternary complex with the two exchanged substrates bound simultaneously. However, recent structural and functional data have demonstrated that the mitochondrial ADP/ATP carrier works as a monomer and has a single substrate binding site, which cannot be reconciled with a sequential kinetic mechanism. Here, we study the kinetic properties of the human mitochondrial ADP/ATP carrier by using proteoliposomes and transport robotics. We show that the Km/Vmax ratio is constant for all of the measured internal concentrations. Thus, in contrast to earlier claims, we conclude that the carrier operates with a ping‐pong kinetic mechanism in which substrate exchange across the membrane occurs consecutively rather than simultaneously. These data unite the kinetic and structural models, showing that the carrier operates with an alternating access mechanism.

## Graphical abstract

The human mitochondrial ADP/ATP carrier (SLC25A4) operates with a ping‐pong kinetic mechanism in which one substrate is imported before the other substrate is exported.

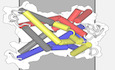

## Introduction

The SLC25 mitochondrial carrier family of transport proteins is responsible for the translocation of a diverse range of compounds across the impermeable mitochondrial inner membrane, linking cytosolic and mitochondrial metabolism and supporting important cellular processes (Kunji *et al*, 2020). In total, 53 different SLC25 members are encoded by the human nuclear genome, most of which transport substrates, such as inorganic ions, co‐factors, nucleotides, amino acids, fatty acids, protons and di‐ and tri‐carboxylates (Ruprecht & Kunji, 2020). Considering their crucial roles in physiology, it is not surprising that an increasing number of pathologies have been associated with the absence or dysfunction of SLC25 members (Kunji *et al*, 2020).

The transport mechanism of mitochondrial carriers has been described by two kinetic models: sequential or ping‐pong (Palmieri *et al*, 1993). In the sequential mechanism, the two exchanged substrates must bind to the carrier, leading to the formation of a ternary complex. The other kinetic model is a ping‐pong or double displacement mechanism, where one substrate is transported first, dissociating from the substrate binding site before a second substrate binds for transport in the opposite direction. These two transport mechanisms can be discriminated by two‐reactant initial‐velocity studies by varying both the internal and external substrate concentrations (Cleland, 1963a, 1963b, 1963c). In case of a sequential mechanism, the Km/Vmax ratio decreases with increasing concentration of the counter‐substrate, leading to convergence in Lineweaver–Burk plots (Appendix Fig S1; Cleland, 1970). For a ping‐pong kinetic mechanism, the ratio of Km/Vmax is independent of the concentration of the counter‐substrate, meaning that the Lineweaver–Burk plots are parallel (Appendix Fig S1; Cleland, 1970). A sequential kinetic mechanism has been proposed for the majority of the mitochondrial carriers, including the mitochondrial bovine oxoglutarate (Indiveri *et al*, 1991a), phosphate (Stappen & Kramer, 1994) and aspartate/glutamate carriers (Dierks *et al*, 1988), as well as the rat mitochondrial ornithine (Indiveri *et al*, 1994), dicarboxylate (Indiveri *et al*, 1989a, 1989b), tricarboxylate (Bisaccia *et al*, 1990, 1993), and ADP/ATP carriers (Duyckaerts *et al*, 1980; Barbour & Chan, 1981). The only mitochondrial carrier suggested to operate by a ping‐pong mechanism is the rat carnitine/acylcarnitine carrier (Indiveri *et al*, 1991b). These analyses, in combination with several biochemical and biophysical studies, suggested that all mitochondrial carriers, including the ADP/ATP carriers, exist and function as homodimers (Brandolin *et al*, 1980; Hackenberg & Klingenberg, 1980; Block *et al*, 1982; Hatanaka *et al*, 1999; Trezeguet *et al*, 2000; Huang *et al*, 2001; Dyall *et al*, 2003; Postis *et al*, 2005). Mechanistic models were proposed, where the two protomers of the dimer were in different states, one open to the matrix and the other open to the intermembrane space (Palmieri *et al*, 1993). Upon binding of both substrates, the protomers would open up to the other side of the membrane to transport the substrates.

However, the dimer model for mitochondrial carriers was challenged when the first structural information for the mitochondrial ADP/ATP carrier became available. The projection structure of the yeast ADP/ATP carrier, inhibited by atractyloside in the cytoplasmic‐open conformation, clearly demonstrated that the carrier is a structural monomer with threefold pseudo‐symmetry (Kunji & Harding, 2003). The structure consists of a ring of six transmembrane helices arranged around a substrate translocation path through the center of the protein, indicating that it could function as a monomer (Kunji & Harding, 2003). Subsequent atomic‐resolution structures of the bovine and yeast ADP/ATP carriers, inhibited with carboxyatractyloside in the cytoplasmic‐open conformation (Pebay‐Peyroula *et al*, 2003; Ruprecht *et al*, 2014), confirmed a monomeric structural fold. Moreover, a careful analysis of all available crystal forms indicated that there is no conserved dimerization interface (Ruprecht *et al*, 2014). A reassessment of all previously published claims for a homodimer led to the identification of a large number of technical issues that contributed to an incorrect assignment of the oligomeric state of the carriers (Kunji & Crichton, 2010; Kunji & Ruprecht, 2020). Recently, the structure of the matrix‐open conformation of the mitochondrial ADP/ATP carrier, inhibited by bongkrekic acid, was solved (Ruprecht *et al*, 2019). The structure provides support for an alternating access mechanism in which a central substrate binding site is made available to one side of the membrane or the other by the formation and disruption of gates on either side of the carrier (Robinson *et al*, 2008). The matrix gate, consisting of the matrix salt bridge network and glutamine braces, forms when the carrier is in the cytoplasmic‐open state and is disrupted in the matrix‐open state (Pebay‐Peyroula *et al*, 2003; Ruprecht *et al*, 2014). Conversely, the cytoplasmic gate, comprising the cytoplasmic salt bridge network and tyrosine braces, forms in the matrix‐open state and is disrupted in the cytoplasmic‐open state (King *et al*, 2016; Ruprecht *et al*, 2019). The carrier is the most dynamic transport protein found to date, having three domains, each containing a core and a gate element, meaning that there are six moving parts in total (Ruprecht *et al*, 2019). When cycling between states, the shape and surface of the carrier change dramatically, excluding the possibility of dimerization (Ruprecht *et al*, 2019). Using different computational and experimental methods, only a single substrate binding site, located in the central cavity, has been detected (Kunji & Robinson, 2006; Robinson & Kunji, 2006; Robinson *et al*, 2008; Ruprecht *et al*, 2019; Mavridou *et al*, 2022). Thus, the current structural mechanism is inconsistent with the sequential mechanism suggested by the earlier kinetic studies (Duyckaerts *et al*, 1980; Barbour & Chan, 1981).

Here, we study the kinetic mechanism of the human mitochondrial ADP/ATP carrier isoform 1 (SLC25A4), also called adenine nucleotide translocase ANT1. The carrier was expressed in the mitochondrial inner membrane of *Saccharomyces cerevisiae*, purified, and reconstituted into liposomes. We used robotic transport assays to obtain the initial rates in a range of controlled substrate gradients, and we conclude that the carrier operates with a ping‐pong kinetic mechanism, reconciling the structural and kinetic mechanisms in favor of the carrier functioning as a monomer.

## Results and Discussion

The establishment of a kinetic mechanism for a transporter is critically dependent on the accurate determination of the initial transport rates for a range of substrate gradients. Most commonly, fixed time intervals are used to get estimates of the transport rates. There are, however, several issues with this approach. In the case of low uptake rates, the substrate accumulation over the time interval could be too small to determine the rate accurately and in the case of high uptake rates, a significant deviation from linearity could occur relatively quickly, leading to an underestimation of the rate. Another problematic aspect is the determination of the zero‐time point, which often follows a different experimental procedure from the other time points, potentially leading to a systematic error. A further complication is that often stop buffers are used, which contain specific or generic inhibitors, meaning that the transport kinetics are intertwined with inhibitor kinetics.

**Figure 1:**
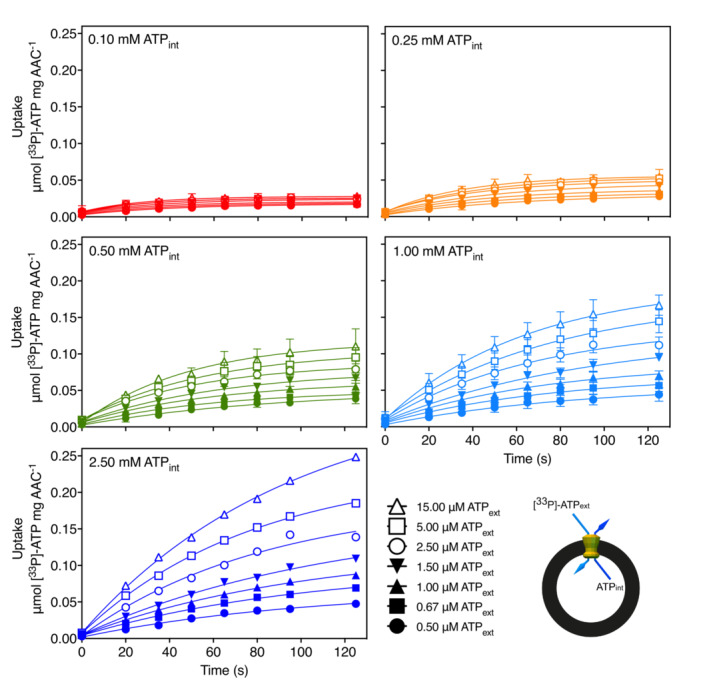
Uptake curves of the transport catalyzed by reconstituted human ADP/ATP carrier Proteoliposomes containing human ADP/ATP carrier were loaded with either 0.10 mM (red traces), 0.25 mM (orange traces), 0.50 mM (green traces), 1.00 mM (light blue traces), or 2.50 mM (dark blue traces) ATP (ATP_int_), and transport was initiated by the externally added radiolabeled [^33^P]‐ATP (ATP_ext_) at either 0.50 μM (filled circles), 0.67 μM (filled squares), 1.00 μM (filled upward triangles), 1.50 μM (filled downward triangles), 2.50 μM (open circles), 5.00 μM (open squares), or 15.0 μM (open upward triangles). The data were fitted to equation 1. A schematic representation of the transport assays is shown. Data information: The data are represented by the average and standard deviation of three biological repeats (each the average of three technical repeats) for the assays with 0.10, 0.25, 0.5, and 1.0 mM of internal ATP, and one biological repeat (based on the average of three technical repeats) for the 2.50 mM internal ATP assay. Source data are available online for this figure.

Here, we have taken a different approach by recording complete uptake curves for the tested concentration gradients with transport robotics. For this purpose, the human ADP/ATP carrier SLC25A4 was expressed recombinantly in the mitochondrial inner membrane of *Saccharomyces cerevisiae* (King & Kunji, 2020; Jaiquel Baron *et al*, 2021), purified in stabilizing buffers containing lauryl maltose neopentyl glycol and cardiolipin, and reconstituted into liposomes. To determine the initial uptake rates at different substrate gradients, we carried out ATP/ATP homo‐exchange experiments. We have previously shown that ATP and ADP bind to the same site in the mitochondrial ADP/ATP carrier and that the transport steps are fully reversible (Mavridou *et al*, 2022). The use of the same substrate internally and externally prevents the build‐up of a membrane potential over the course of the assay, which would be an unnecessary complicating factor. We loaded proteoliposomes of reconstituted human ADP/ATP carrier with either no substrate, which served as a background control, or with five different internal concentrations (0.10, 0.25, 0.50, 1.00, or 2.50 mM ATP), and initiated transport with seven different concentrations of radiolabeled [^33^P]‐ATP (Fig 1).

The initial rates for each of the substrate gradients were obtained by fitting the uptake curves with the following equation (Appendix):

(1) $$Q_{i}=\beta \;\left(1-e^{-\frac{k}{\beta }\left(t-d\right)}\right)$$

Where $Q_{i}$ is the quantity of radiolabeled substrate on the inside of the proteoliposomes, β is the total quantity of substrate exchanged at equilibrium, k is the initial influx rate of radiolabeled substrate, t the exchange period, and d is the time delay due to filtration. For the initial phase, the equation approximates as:

(2) $$Q_{i}=k\;\left(t-d\right)$$

**Figure 2:**
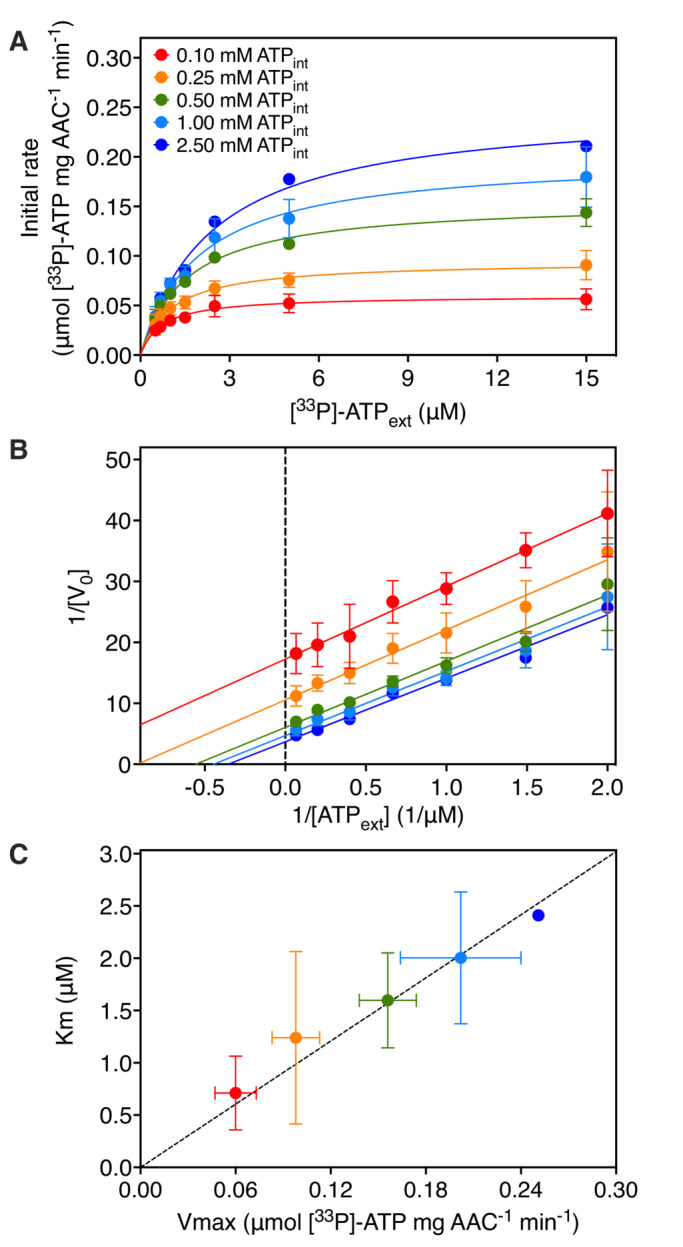
Two‐substrate analysis of transport catalyzed by the mitochondrial ADP/ATP carrier Initial rates were estimated by fitting the data with the model in equation 1. Michaelis–Menten plots of 0.10 mM (red traces), 0.25 mM (orange traces), 0.50 mM (green traces), 1.00 mM (light blue traces), and 2.50 mM (dark blue traces) internally loaded ATP.Lineweaver–Burk analysis of [^33^P]‐ATP/ATP homo‐exchange (same color scheme as A).Km plotted against Vmax for the various substrate gradients. The kinetic parameters were determined by fitting the Michaelis–Menten curves through iteration (same color scheme as A). Data information: The data are represented by the average and standard deviation of three biological repeats (each the average of three technical repeats) for all internal concentrations but 2.50 mM ATP, which is the average of three technical repeats. Source data are available online for this figure.

Thus, the quantity of internal radiolabeled substrate increases linearly with time during the initial exchange. By fitting the data with equation 1, accurate estimates of the initial uptake rates, given by k, can be obtained at both high and low concentrations, as each data point of the uptake curve was determined with the same procedure (Fig 1). In this way, the time frame in which the rate is linear can also be determined, and nonlinear data can be excluded. The initial rates obtained using equation 1 were then used to construct Michaelis–Menten curves and Lineweaver–Burk plots for each experiment (Fig 2A and B).

The Lineweaver–Burk lines for different internal concentrations appear to be parallel upon visual inspection (Fig 2B), consistent with a ping‐pong kinetic mechanism (Cleland, 1970). In general, the disadvantage of Lineweaver–Burk representations is that they disproportionately depend on the less accurately determined points obtained at low concentrations, whereas the more accurately determined points at high concentrations cluster and thus are less valuable in determining the slope of the lines. A better approach is to obtain the apparent Km and Vmax values by fitting the Michaelis–Menten curves (Fig 2A) and to plot the kinetic parameters against each other (Fig 2C). Analyzed in this way, the data show that the Km/Vmax ratio for all of the measured internal concentrations is constant within experimental error (Fig 2C), demonstrating conclusively that the human mitochondrial ADP/ATP carrier operates with ping‐pong kinetic mechanism.

In contrast, the vast majority of mitochondrial carriers have previously been proposed to operate with a sequential mechanism, in which the two substrates must be bound to the carrier at the same time for exchange to occur (Palmieri *et al*, 1993). In particular, the rat mitochondrial ADP/ATP carrier was proposed to have a sequential mechanism using kinetic analysis with isolated mitochondria (Duyckaerts *et al*, 1980; Barbour & Chan, 1981). Although the sequential kinetic mechanism had no molecular detail, it was generally assumed that the carriers were homodimers, where one protomer would bind ADP and the other ATP for transport in opposing directions. This mechanism would also account for the observation that ADP and ATP are exchanged in equimolar amounts (Heldt *et al*, 1965; Pfaff *et al*, 1965). However, there are several complicating factors when mitochondria are used for kinetic studies. First, mitochondrial ADP and ATP concentrations change substantially depending on the metabolic state. Second, it is difficult to control the leakage of endogenous nucleotides, which will skew the concentration gradients. Third, there are a large number of mitochondrial enzymes that can interconvert ADP and ATP, such as ATP synthase, creatine kinase, hexokinase, and ABC transporters, meaning that the nucleotide species is not defined. Fourth, in the context of the mitochondrion, adenine nucleotides can chelate magnesium ions, but the ADP/ATP carrier transports only free nucleotides (Pfaff & Klingenberg, 1968). Fifth, mitochondria contain not only different isoforms of mitochondrial ADP/ATP carriers but also ATP‐Mg/phosphate carriers (Austin & Aprille, 1984; Fiermonte *et al*, 2004), CoA transporters (Fiermonte *et al*, 2009) and thiamine pyrophosphate carriers (Lindhurst *et al*, 2006), all capable of transporting ADP and/or ATP, albeit with different kinetics. Thus, mitochondria represent an extremely complex system with which to perform these kinetic analyses.

**Figure 3:**
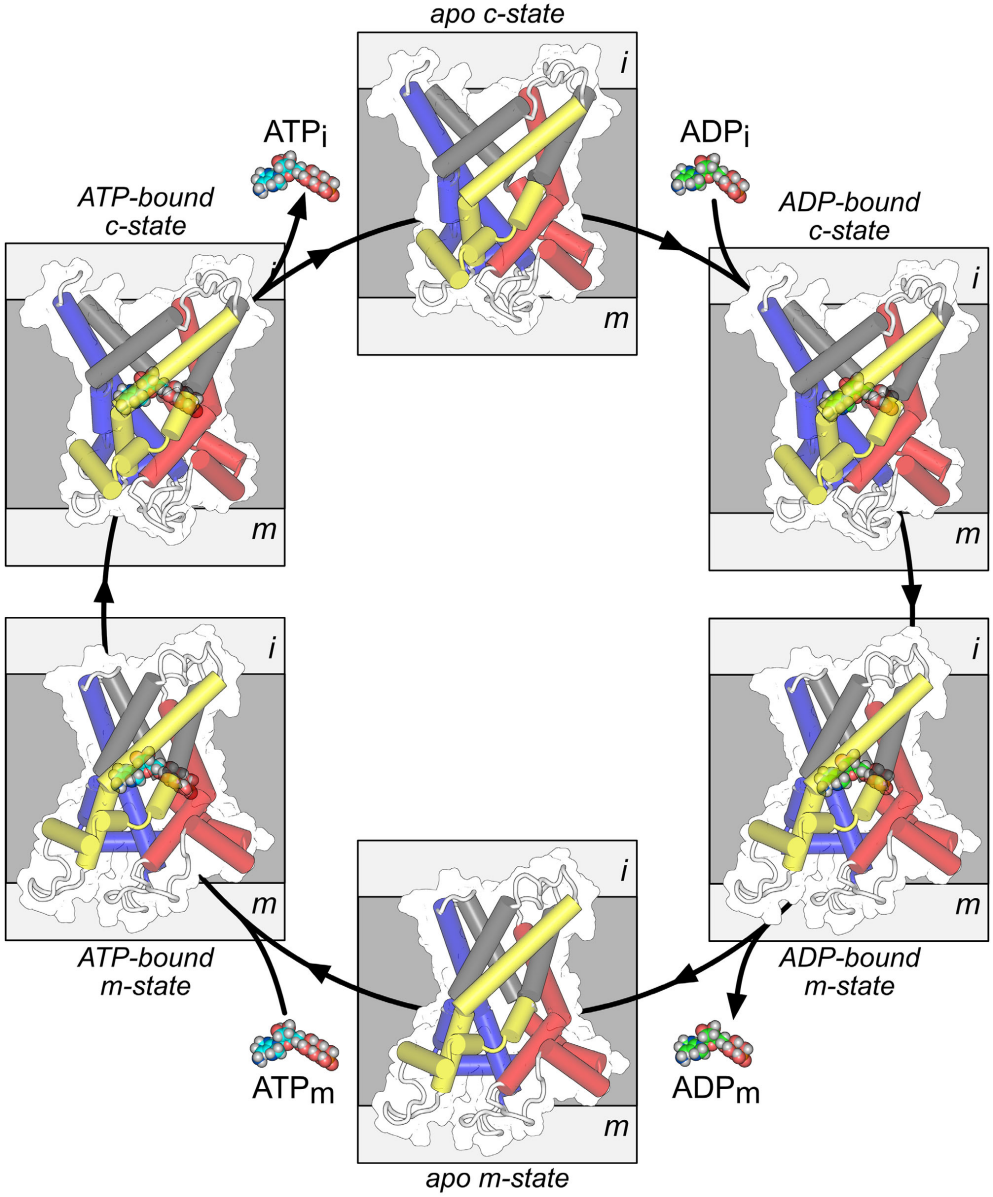
Schematic representation of the ping‐pong mechanism of the mitochondrial ADP/ATP carrier Conformational changes between the cytoplasmic‐open state (c‐state) and matrix‐open state (m‐state) with the helices shown as cylinders, viewed laterally from the membrane. The occluded states have not been resolved structurally and are not shown. The three core elements are colored in blue, yellow, and red, respectively, and the three gate elements in gray. The substrates ADP and ATP are shown in green and cyan sphere representations, respectively. Abbreviations: m for mitochondrial matrix; i for intermembrane space.

Here, we have used a straightforward experimental system using proteoliposomes wherein the internal and external concentrations of the transported substrate species can be tightly controlled. By using robotics, we have recorded whole uptake curves for a large number of defined substrate gradients, which can be fitted to provide an accurate determination of the initial rates. We have shown that the human mitochondrial ADP/ATP carrier operates with a ping‐pong mechanism, which agrees with an alternating access mechanism in which a transported substrate dissociates from the protein before a counter‐substrate binds for transport in the other direction (Fig 3). The data presented in this paper do not directly address the question of the oligomeric state of the ADP/ATP carrier. However, they do provide further support for the notion that the carrier functions as a monomer with a single substrate binding site, consistent with a large number of structural and functional data gathered over recent years.

First of all, the mitochondrial ADP/ATP carrier has a monomeric structural fold (Kunji & Harding, 2003; Pebay‐Peyroula *et al*, 2003; Ruprecht *et al*, 2014, 2019), and there are no sequence or structural data that would support a plausible dimerization interface (Kunji & Crichton, 2010; Ruprecht *et al*, 2014). When the detergent/lipid micelle is properly accounted for, the carrier is monomeric in sizing experiments (Bamber *et al*, 2006; Kunji *et al*, 2008; Kunji & Crichton, 2010; Crichton *et al*, 2013), even in the mildest detergents (Bamber *et al*, 2007b). The conformational changes in the transport cycle involve large movements of six mobile elements that change the shape and surface of the carrier dramatically during the transport cycle (Ruprecht *et al*, 2019), precluding any stable interactions with other carriers or other mitochondrial proteins. The monomeric carrier has all of the properties required for a functional transport protein (Ruprecht *et al*, 2019; Ruprecht & Kunji, 2020), and there are also no negative dominant effects between co‐expressed carriers, demonstrating that the carrier functions as a monomer (Bamber *et al*, 2007a). The two salt bridge networks have fully explained the observed equimolar exchange of adenine nucleotides (Pfaff *et al*, 1965), as they can only be disrupted upon binding of substrate to induce conformational changes (Robinson *et al*, 2008), negating the need for a dimeric model. Thus, all sequence, structural, and functional data point toward the carrier being active as a monomer.

Second, for the sequential mechanism to be valid, there would have to be two substrate binding sites in the functional unit, but all current evidence points to the presence of only one. Using chemical and distance constraints, three contact points that define the substrate specificity of mitochondrial carriers were discovered in the central cavity on the even‐numbered helices (Kunji & Robinson, 2006; Robinson & Kunji, 2006). Later, these contact points were also found to be the hinge points between the core and gate elements in all three domains, coupling substrate binding to the structural mechanism (Ruprecht *et al*, 2019). Since mitochondrial carriers are threefold pseudo‐symmetric, asymmetric binding sites must have evolved to bind the asymmetric substrates (Robinson *et al*, 2008). Using this principle, a single substrate binding site in the central cavity was found, which had a complementary chemistry to the substrates (Robinson *et al*, 2008). A comparison of the matrix‐open and cytoplasmic‐open structures clearly shows that the same central binding site is accessible to substrates via a water‐filled cavity, meaning that a bound substrate could be transported from one side of the membrane to the other via an occluded state (Ruprecht *et al*, 2019). Recently, direct experimental evidence for a single substrate binding site has been obtained by using thermostability shift assays (Mavridou *et al*, 2022). These analyses used the observation that a concentration‐dependent thermostability shift occurs in the presence of ADP or ATP (Majd *et al*, 2018; Mavridou *et al*, 2022). Single alanine replacement mutants of the translocation pathway were screened in the presence of these substrates. Five residues were found to be essential and another six important for substrate binding, and all of them cluster together in the central cavity, indicating a single substrate site (Mavridou *et al*, 2022). The fact that a single mutation can abolish the shift completely provides further independent proof that there is only one substrate binding site. Furthermore, all shifts were similar for both ADP and ATP, demonstrating that they both bind to the same site with the same chemistry (Mavridou *et al*, 2022). Thus, all evidence points toward a single substrate binding site in the central cavity of the carrier, which is alternately accessible to either side of the membrane.

The data presented in this paper support a ping‐pong kinetic mechanism, which is in agreement with our proposed structural mechanism (Fig 3). Based on the related structural and functional properties, it is likely that other SLC25 mitochondrial carriers have a similar transport mechanism (Ruprecht & Kunji, 2020), but more work is required to analyze their kinetic mechanisms in order to address the aforementioned discrepancies in the literature.

## Materials and Methods

### Expression of human ADP/ATP carrier in *Saccharomyces cerevisiae*

The expression of properly folded, full‐length mammalian ADP/ATP carrier in yeast cells is known to be problematic (Hatanaka *et al*, 2001; Jaiquel Baron *et al*, 2021). The gene for the truncated human ADP/ATP carrier isoform 1 (hAAC Δ1‐10) was cloned into a derivative of the pYES2/CT vector (Invitrogen) in which the galactose promoter has been replaced by the constitutive promoter pMIR of the *S. cerevisiae* phosphate carrier, and transformed into yeast strain W303.1B (King & Kunji, 2020; Jaiquel Baron *et al*, 2021). The truncation leads to increased expression levels and does not interfere with the known functions of the carrier (Jaiquel Baron *et al*, 2021). Transformants were selected on Sc‐Trp + 2% (w/v) glucose plates. For large‐scale expression, a preculture of cells grown in Sc‐Trp + 2% (w/v) glucose was inoculated into 100 L of YEPG medium in an Applikon Pilot Plant 140 L bioreactor. Cells were grown at 30°C for 24 h and harvested by centrifugation (4,000 *g*, 20 min, 4°C). Crude mitochondria were prepared using a bead mill (Dyno‐Mill Multilab, Willy A. Bachofen AG) by established methods (King & Kunji, 2020).

### Preparation of lipid for protein purification

Tetraoleoyl cardiolipin (18:1) powder (Avanti Polar Lipids) was solubilized in 10% (w/v) lauryl maltose neopentyl glycol (Anatrace) by vortexing for 4 h to give 10 mg ml^−1^ lipid in a 10% (w/v) detergent stock. The stocks were stored in liquid nitrogen.

### Purification of the human ADP/ATP carrier with nickel affinity chromatography

Protein was prepared as described previously (Jaiquel Baron *et al*, 2021). Crude mitochondria were solubilized in 1% (w/v) lauryl maltose neopentyl glycol, EDTA‐free protease inhibitor tablets (Roche), 20 mM imidazole and 150 mM NaCl for 1 h by rotation at 4°C. The insoluble material was separated from the soluble fraction by centrifugation (200,000 *g*, 45 min, 4°C). Nickel sepharose slurry (0.7 ml resin per 1 g of crude mitochondria; GE Healthcare) was added to the soluble fraction; the mixture was stirred at 4°C for 1 h. The nickel resin was harvested by centrifugation (100 *g*, 10 min, 4°C), transferred to a proteus 1‐step batch midi spin column (Generon), and washed by centrifugation (100 *g*, 15 min, 4°C) with 30 column volumes (CV) buffer A (20 mM HEPES pH 7.0, 150 mM NaCl, 40 mM imidazole, 0.2 mg ml^−1^ tetraoleoyl cardiolipin/0.2% (w/v) lauryl maltose neopentyl glycol); followed by 10 CV buffer B (20 mM HEPES pH 7.0, 50 mM NaCl, 0.2 mg ml^−1^ tetraoleoyl cardiolipin/0.2% (w/v) lauryl maltose neopentyl glycol). The nickel resin was resuspended in an equal volume of buffer (0.7 ml) and incubated with 20 mM imidazole, 30 μg factor Xa protease (NEB) and 5 mM CaCl_2_ overnight at 4°C with rotation. The protein was eluted by centrifugation (500 *g*, 2 min, 4°C), and imidazole was removed using a midi PD‐10 desalting column (GE Healthcare) at 4°C. Protein concentration in the elution fractions was measured by spectrometry (NanoDrop Technologies) at 280 nm (extinction coefficient; 47,120 M^−1^ cm^−1^, protein mass; 32,380 Da). The purity and stability of the final sample were assessed by gel electrophoresis and thermostability shift assays, respectively.

### Reconstitution of protein into liposomes

Liposomes consisting of egg L‐α‐phosphatidylcholine and tetraoleoyl cardiolipin (Avanti Polar Lipids) were dried under a stream of nitrogen. A unit of reconstitution consisted of 12.6 mg of total lipids (12 mg egg L‐α‐phosphatidylcholine and 0.6 mg tetraoleoyl cardiolipin) and 50 μg protein in a total volume of 1.25 ml (Jaiquel Baron *et al*, 2021). This sample was enough for transport assays with a single internal concentration and seven external concentrations. Lipids were rehydrated in 20 mM HEPES pH 7.0 and 50 mM NaCl by vortexing. Samples were kept on ice, and the lipids were solubilized by the addition of the detergent pentaethylene glycol monododecyl ether (C_10_E_5_) until full clarification. ATP was added to the reconstitution mixture at the desired concentrations and the detergent was removed by SM‐2 bio‐beads (Bio‐Rad). Five additions of bio‐beads were made to the master mix every 20 min with inversion at 4°C: the first four had 60 mg bio‐beads, and the final one 480 mg per reconstitution unit. The sample was incubated overnight at 4°C using inversion. Bio‐beads were removed by passage through empty micro‐bio spin columns (Bio‐Rad), and proteoliposomes were subsequently extruded by 11 passages through a 1.0‐μm filter (Millipore). PD10 desalting columns (GE Healthcare) equilibrated with transport buffer (20 mM HEPES pH 7.0 and 50 mM NaCl) were used to remove external substrate. Desalted proteoliposomes (1.5 ml) were diluted eightfold in the aforementioned buffer before performing transport.

### Transport assays

Transport assays were performed using the Hamilton MicroLab Star robot (Hamilton Robotics Ltd). Diluted proteoliposomes (100 μl) were loaded into the wells of a MultiScreenHTS + HA 96‐well filter plate (pore size 0.45‐μm, Millipore). Uptake of radiolabeled [^33^P]‐ATP (Hartmann Analytic) was initiated by the addition of 100 μl buffer containing 0.50, 0.67, 1.00, 1.50, 2.50, 5.00, or 15.00 μM [^33^P]‐ATP, all prepared to 5.0 GBq mmol^−1^. Uptake was stopped at 0, 20, 35, 50, 65, 80, 95, and 125 s by filtration and washing with 3 × 200 μl ice‐cold buffer (20 mM HEPES, pH 7.0 and 50 mM NaCl). Plates were dried overnight, 200 μl MicroScint‐20 (Perkin Elmer) added, and levels of radioactivity determined using a TopCount scintillation counter (Perkin Elmer). Uptake was normalized to quantified protein, and curves were fitted in Prism (GraphPad) using an exponential rise to max model based on equation 1. Initial rates were given by *k*, and apparent Km and Vmax values were determined by fitting the Michaelis–Menten curves in Prism.

### Protein quantification

Pure protein standards and desalted proteoliposome samples were run on SDS‐polyacrylamide gels (4–12% gradient gels, mPAGE, Merck) at 180 V. Gels were incubated in fixing solution (40% ethanol, 10% acetic acid) at room temperature for 2 h, followed by overnight incubation at 4°C in 1 × Flamingo fluorescent gel stain (Bio‐Rad). Imaging of the gels was performed with the Amersham Typhoon (GE Healthcare)—Cy2 emission filter; 500–550 PMT (V); pixel size of 25 μm. The one‐dimensional gel analysis of the ImageQuant TL software (Toolbox v8.1) was used to analyze the images. The rolling ball method with a radius of 200 was used for background subtraction. Band volumes were used to calculate the amount of protein in the liposome samples relative to the standard curve (ranging from 10 to 100 ng).

## Author contributions

**Camila Cimadamore‐Werthein:** Formal analysis; investigation; visualization; methodology; writing – original draft; writing – review and editing. **Stephany Jaiquel Baron:** Formal analysis; investigation; visualization; methodology; writing – review and editing. **Martin S King:** Conceptualization; formal analysis; investigation; visualization; methodology; writing – original draft; writing – review and editing. **Roger Springett:** Software; formal analysis; methodology; writing – review and editing. **Edmund RS Kunji:** Conceptualization; formal analysis; supervision; funding acquisition; visualization; writing – original draft; project administration; writing – review and editing.

## Disclosure and competing interests statement

The authors declare that they have no conflict of interest.

## Supporting information



Appendix S1

Source Data for Figure 1

Source Data for Figure 2

## Data Availability

This study includes no data deposited in external repositories.
