# Supplementary material for: Human mitochondrial ADP/ATP carrier SLC25A4 operates with a ping‐pong kinetic mechanism
Source: EMBO Rep. 2023 Jun 6;24(8):e57127. doi: 10.15252/embr.202357127 (PMC10398649; doi:10.15252/embr.202357127)
Supplement: Supplementary file 1 — Appendix S1 [file EMBR-24-e57127-s004.pdf]

Appendix

Table of contents

Appendix Figure S1 (page 2)

Fitting of uptake curves to obtain the best estimate of the initial rates (page 3)

Appendix Figure

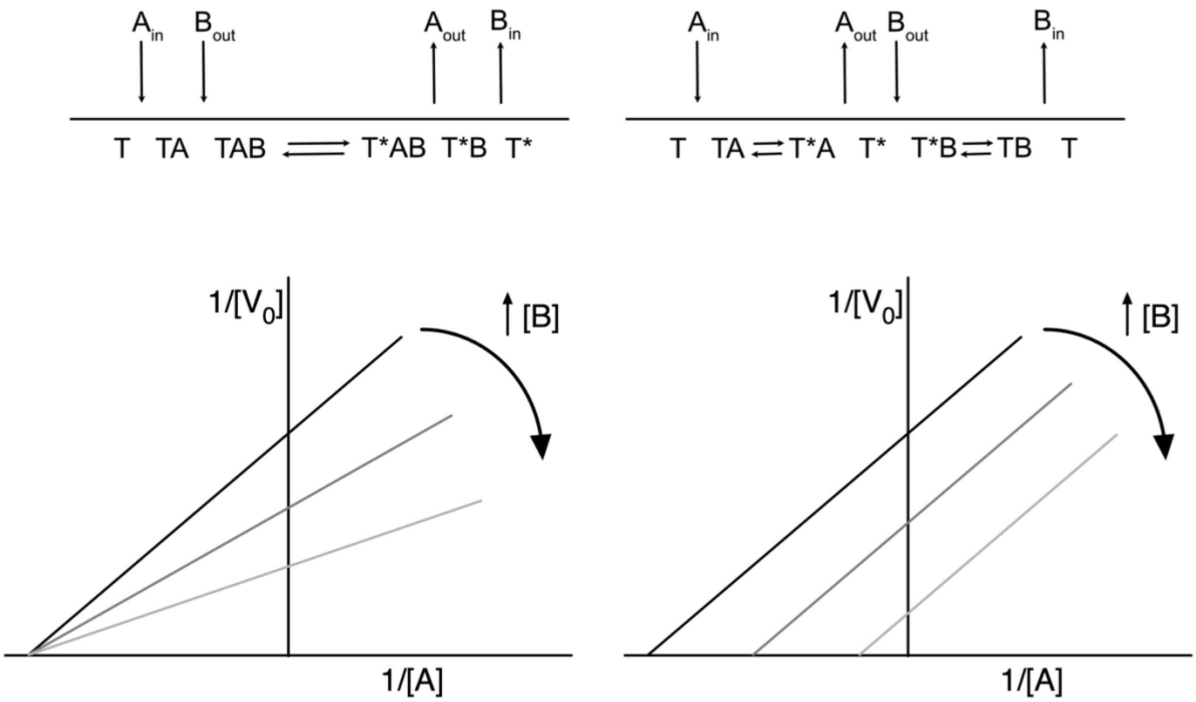

Appendix Figure S1: Schematic and experimental differentiation of (left) sequential and (right) ping-pong kinetic mechanisms by two-reactant initial-velocity studies. A and B are the two exchanged substrates, T and T* represent the two conformational states of the transporter.

Fitting of uptake curves to obtain the best estimate of the initial rates

Considerations

Initially, each uptake experiment contains proteoliposomes with reconstituted ADP/ATP carrier with unlabeled substrate on the inside and radiolabeled substrate on the outside at defined concentrations. The exchange of substrates by the carrier occurs with a 1:1 stoichiometry, resulting in the accumulation of radiolabeled substrate on the inside and unlabeled substrate on the outside. At defined time intervals the external substrate is removed through filtration and the amount of accumulated radiolabeled substrate is determined through scintillation counting. The uptake reaches equilibrium when the same amount of radiolabeled substrate is imported into the proteoliposomes as is exported from them.

Nomenclature and Units

We use P to indicate unlabeled substrate, Q to indicate radiolabeled substrate and T for the total. The subscript o indicates outside, whereas subscript i indicates inside the proteoliposomes.

Symbol	Meaning
Q_o	Radiolabeled substrate on outside
Q_i	Radiolabeled substrate on inside
P_o	Unlabeled substrate on outside
P_i	Unlabeled substrate on inside
T_o	Total substrate on outside
T_i	Total substrate on inside

Square brackets around a symbol denote concentration e.g. $[Q_o]$ is the concentration (in µM) of radiolabeled substrate on the outside. The symbol alone denotes quantity e.g. Q_o is the quantity (in pmol) of radiolabeled substrate on the outside. V_o and V_i are the volume of the inside and outside. Concentrations are in µM, quantities in pmol and volumes are in µL. The quantities and concentrations are related by the relevant volume e.g. $V_o[Q_o]=Q_o$. Due to the 1:1 exchange stoichiometry, the sum of the radiolabeled and unlabeled substrate (total substrate T) on the inside and outside is constant and equal to the initial substrate concentrations on each side, e.g.

	$Q_o + P_o = T_o$<br>$Q_i + P_i = T_i$	**Equation 1**

where T_o and T_i are the total quantity of substrate on the outside and inside, respectively.

Transport

The catalyzed exchange reaction is

	$Q_o+P_i \leftrightarrow Q_i+P_o$	**Equation 2**

The ΔG for this reaction is

	$\Delta G = \Delta G^o + RT \,\mathrm{Ln}\left(\frac{[Q_i][P_o]}{[Q_o][P_i]}\right)$	**Equation 3**

Where R is the gas constant and T is the temperature in Kelvin. The $\Delta G°$ is 0 because no chemical reaction occurs. Thus, equilibrium will be reached when ΔG=0 or when

	$[Q_i][P_o]=[Q_o][P_i]$	**Equation 4**

By multiplying both sides of Equation 4 by V_oV_i, Equation 4 can be rewritten:

	$Q_iP_o=Q_oP_i$	**Equation 5**

Using Equation 1 to express Q_o and P_i in terms of T_o and P_o, and in terms of T_i and Q_i, respectively, gives

	$Q_iP_o=(T_o-P_o)(T_i-Q_o)$	**Equation 6**

Which can be rearranged to give:

	$T_oT_i=T_oQ_i+P_oT_i$	**Equation 7**

Given the initial conditions in which there is no radiolabeled substrate on the inside and only radiolabeled substrate on the outside, then the quantity of radiolabeled substrate on the inside will equal the quantity of unlabeled substrate on the outside by exchange and thus P_o=Q_i. In this case, Equation 7 can be rewritten

	$Q_i = \frac{T_o T_i}{T_o + T_i}$	**Equation 8**

Where Q_i is the quantity of radiolabeled ATP on the inside at equilibrium, that is the total amount of ATP exchanged.

Kinetics

The rate of substrate exchange by the ADP/ATP carrier is *k* picomoles of substrate per second and does not change over the course of the experiment, because the internal and external concentrations of total substrate are constant, and radiolabeled and unlabeled substrate have identical interactions with the carrier. The value of *k* depends on the V_{max} of the carrier, the internal and external concentration of substrate through the internal and external K_M, respectively. The rate of influx of radiolabeled and unlabeled substrate (k_Q^{influx} and k_P^{influx}, respectively) depends on the relative concentration of radiolabeled and unlabeled substrate on the outside, and are given by:

	$k_Q^{\text{influx}} = k[Q_o]/[T_o]$<br>$k_P^{\text{influx}} = k[P_o]/[T_o]$	**Equation 9**

and likewise for the efflux of radiolabeled and unlabeled substrate, which depends on the concentration of radiolabeled and unlabeled substrate on the inside, e.g.

	$k_Q^{\text{efflux}} = k[Q_i]/[T_i]$<br>$k_P^{\text{efflux}} = k[P_i]/[T_i]$	**Equation 10**

The initial influx of radiolabeled substrate is given by *k* because the initial concentration of substrate on the outside is [T_o]. Note that these rates are in picomoles per second and the effect of this flux on the internal and external concentrations will depend on the volume of each compartment. The rate of accumulation of radiolabeled substrate inside the vesicle is equal to the difference between the influx and the efflux of radiolabeled substrate, e.g.

	$V_i \frac{d[Q_i]}{dt} = -k[Q_i]/[T_i] + k[Q_o]/[T_o]$	**Equation 11**

The total quantity of radiolabeled substrate is constant and, due to the starting conditions of the experiment, equal to $V_o[T_o]$, hence

	$V_o[T_o] = V_i[Q_i] + V_o[Q_o]$	**Equation 12**

If [Q_o] from Equation 12 is substituted in Equation 11 then

	$\frac{d[Q_i]}{dt} = -\frac{k}{\beta}[Q_i] + \frac{k}{V_i}$	**Equation 13**

Where

	$\beta = \frac{[T_i]V_i[T_o]V_o}{[T_i]V_i + [T_o]V_o} = \frac{T_i T_o}{T_i + T_o}$	**Equation 14**

And has units of pmol. This can be solved to give

	$[Q_i] = \frac{\beta}{V_i}\left(1 - e^{-\frac{k}{\beta}t}\right)$ $Q_i = \beta\left(1 - e^{-\frac{k}{\beta}t}\right)$	**Equation 15**

From here, it can be seen that *β* is the total quantity of substrate exchanged (see Equation 8). This is referred to as the exponential solution. For short time intervals, Equation 15 approximates as

	$Q_i = kt$	**Equation 16**

and the quantity of internal radiolabeled substrate increases linearly with time. If a delay in the separation of the external and internal radiolabel due to filtration occurs, a delay factor *d* can be introduced, where *t* becomes *t-d*, as used in the main text.
