# Supplementary material for: Human mitochondrial ADP/ATP carrier SLC25A4 operates with a ping‐pong kinetic mechanism
Source: EMBO Rep. 2023 Jun 6;24(8):e57127. doi: 10.15252/embr.202357127 (PMC10398649; doi:10.15252/embr.202357127)
Supplement: Supplementary file 4 [file EMBR-24-e57127-s002.zip › EMBOR202357127_SourceData_Checklist_1680687049_1.pdf]

I have carefully looked at your figures and prepared a detailed list of the figure panels ("Source data checklist") for which we ask source data – see below.

Beside the panel number I note down some keywords such as 'Numerical Data' or 'Image Data' to classify the experiment and data type. This is to help you and our team to assemble and check the respective files.

I would like you to fill out the SourceData checklist and return it when you submit the revised manuscript and provide the requested source data files along with your resubmission.

In case you are not able to provide source data for one or more of the suggested panels, leave a comment in the blue box at the bottom of the page. You are free to upload any file/image to an external repository (for example BioImage or EMPIAR, see also the attached FAQs document) instead of uploading files to the submission platform. If you decide to do so, please leave a comment to indicate the respective accession number(s) in the blue box at the end of the page: please note that the same accession numbers must also be listed in the "data availability" section of your manuscript.

If during the revision process the panel numbering changes, update the panel number in the respective textbox in the column "New Panel #".

If you have generated new panels for which you can provide source data, please add a comment in the blue box at the end of the page and add the source data files in the corresponding figure folder as indicated in the attached FAQs list file.

When you submit the revised version, upload this document as a "Related Manuscript" file. (Please do not change the file name). Further description on how to upload the source data is provided in the attached FAQs.

You can find more information about SourceData at EMBO Press via this link https://www.embopress.org/pb-assets/embo-site/Guide%20for%20SourceData%20Submission-1656066810500.pdf.

Best,
Hannah

--
Hannah Sonntag, PhD
SourceData Scientific Coordinator,
EMBO https://sourcedata.embo.org

SourceData Checklist

Manuscript ID: EMBOR-2023-57127

SD provided	New Panel #	Panel #	
		1A	Numerical data: Concentration measurement
		2B	Numerical data: Concentration measurement
		2C	Numerical data: Concentration measurement
		2D	Numerical data: IC50/Kd/Km calculation
