# Supplementary material for: Human mitochondrial ADP/ATP carrier SLC25A4 operates with a ping‐pong kinetic mechanism
Source: EMBO Rep. 2023 Jun 6;24(8):e57127. doi: 10.15252/embr.202357127 (PMC10398649; doi:10.15252/embr.202357127)
Supplement: Supplementary file 4 [file EMBR-24-e57127-s002.zip › EMBOR202357127V4RPF_no_figs_1684407851_1.pdf]

EMBO reports

Human mitochondrial ADP/ATP carrier SLC25A4 operates with a ping-pong kinetic mechanism

Camila Cimadamore-Werthein, Stephany Jaiquel Baron, Martin King, Roger Springett, and Edmund Kunji
DOI: 10.15252/embr.202357127

Corresponding author(s): Edmund Kunji (ek@mrc-mbu.cam.ac.uk)

Review Timeline:

Submission Date:	6th Mar 23
Editorial Decision:	3rd Apr 23
Revision Received:	15th Apr 23
Editorial Decision:	11th May 23
Revision Received:	15th May 23
Editorial Decision:	16th May 23
Revision Received:	17th May 23
Accepted:	17th May 23

Editor: Ioannis Papaioannou

Transaction Report:

(Note: With the exception of the correction of typographical or spelling errors that could be a source of ambiguity, letters and reports are not edited. Depending on transfer agreements, referee reports obtained elsewhere may or may not be included in this compilation. Referee reports are anonymous unless the Referee chooses to sign their reports.)

1st Editorial Decision 3rd Apr 2023

Dear Prof. Kunji,

Thank you for submitting your research manuscript for consideration by EMBO reports. It has now been seen by three experts in the field, and we have received the full set of their reports, which are included below.

As you will see, referees #1 and #2 are very positive about this study, they acknowledge the significance of the topic and the technical quality of the investigation, they find the manuscript well-written, and they recommend its publication after minor revision. Referee #3, on the other hand, identifies contradictions with the published literature and does not agree with your interpretation of the presented data.

Given the referee reports, we would like to invite you to revise your manuscript with the understanding that the referee concerns (as detailed in their reports) must be addressed and their suggestions taken on board. Please address all referee concerns in a complete point-by-point response. Acceptance of the manuscript will depend on a positive outcome of a second round of review. It is EMBO reports policy to allow a single round of revision only and acceptance or rejection of the manuscript will therefore depend on the completeness of your responses included in the next, final version of the manuscript. If you have any questions or comments, we can also discuss the revisions in a video chat, if you like.

We realize that it is difficult to revise to a specific deadline. In the interest of protecting the conceptual advance provided by the work, we usually recommend a revision within 3 months (July 2nd). Please discuss with me the revision progress ahead of this time if you require more time to complete the revisions.

Please note that you can publish your study either as a Report or as an Article. For Reports, the manuscript should not exceed 27,000 characters (including spaces but excluding Materials & Methods and References) and 5 main plus 5 Expanded View figures. The Results and Discussion sections must be combined, which will help to shorten the manuscript text by eliminating some redundancy that is inevitable when discussing the same experiments twice. For an Article there are no length limitations, but it should have more than 5 main figures and the Results and Discussion sections must be separate. In both cases, the entire Materials and Methods must be included in the main manuscript file.

*****
IMPORTANT NOTE:
We perform an initial quality control of all revised manuscripts before re-review. Your manuscript will FAIL this control and the handling will be DELAYED if the following APPLIES:

1) If a data availability section providing access to data deposited in public databases is missing. If you have not deposited any data, please add a sentence to the data availability section that explains that (see below for more information).

2) If your manuscript contains statistics and error bars based on n=2. Please use scatter plots in these cases. No statistics should be calculated if n=2.

When submitting your revised manuscript, please carefully review the instructions that follow below. Failure to include requested items will delay the evaluation of your revision.
*****

When submitting your revised manuscript, we will require:

1) A .docx formatted version of the manuscript text (including legends for main figures, EV figures and tables). Please make sure that the changes are highlighted to be clearly visible.

2) Individual production quality figure files as .eps, .tif, .jpg (one file per figure).
Please download our Figure Preparation Guidelines (figure preparation pdf) from our Author Guidelines pages https://www.embopress.org/page/journal/14693178/authorguide for more info on how to prepare your figures.

3) A .docx formatted letter INCLUDING the reviewers' reports and your detailed point-by-point responses to their comments. As part of the EMBO Press transparent editorial process, the point-by-point response is part of the Review Process File (RPF), which will be published alongside your paper unless you opt out of this (please see below for further information).

4) A complete author checklist, which you can download from our author guidelines (). Please insert information in the checklist that is also reflected in the manuscript. The completed author checklist will also be part of the RPF (please see below for more information).

5) Please note that all corresponding authors are required to supply an ORCID ID for their name upon submission of a revised manuscript (). Please find instructions on how to link your ORCID ID to your account in our manuscript tracking system in our Author guidelines
()

6) We replaced Supplementary Information with Expanded View (EV) Figures and Tables that are collapsible/expandable online. A maximum of 5 EV Figures can be typeset. EV Figures should be cited as 'Figure EV1, Figure EV2" etc... in the text and their respective legends should be included in the main text after the legends of regular figures.

- For the figures that you do NOT wish to display as Expanded View figures, they should be bundled together with their legends in a single PDF file called *Appendix*, which should start with a short Table of Content. Appendix figures should be referred to in the main text as: "Appendix Figure S1, Appendix Figure S2" etc. See detailed instructions regarding expanded view here:

- Additional Tables/Datasets should be labeled and referred to as Table EV1, Dataset EV1, etc. Legends have to be provided in a separate tab in case of .xls files. Alternatively, the legend can be supplied as a separate text file (README) and zipped together with the Table/Dataset file.

7) Please note that a "Data availability" section at the end of Materials and Methods is now mandatory. In case you have no data that require deposition in a public database, please state so instead of refereeing to the database: "Our study includes no data deposited in public repositories." under the heading "Data availability".
See also). Please note that the Data availability statement is restricted to new primary data that are part of this study.

8) We request authors to consider both actual and perceived competing interests. Please review the new policy () and update your competing interests statement if necessary. Please name this section 'Disclosure and competing interests statement' and place it after the Acknowledgements section.

9) Figure legends and data quantification:
The following points must be specified in each figure legend:

- the name of the statistical test used to generate error bars and P values,
- the number (n) of independent experiments (please specify technical or biological replicates) underlying each data point,
- the nature of the bars and error bars (s.d., s.e.m.)
- If the data are obtained from n {less than or equal to} 2, use scatter plots showing the individual data points.

Discussion of statistical methodology can be reported in the Materials and Methods section, but figure legends should contain a basic description of n, P and the test applied.

See also the guidelines for figure legend preparation:
https://www.embopress.org/page/journal/14693178/authorguide#figureformat

10) We now request publication of original source data with the aim of making primary data more accessible and transparent to the reader. Our source data coordinator will contact you to discuss which figure panels we would need source data for and will also provide you with helpful tips on how to upload and organize the files.

11) Our journal encourages inclusion of *data citations in the reference list* to directly cite datasets that were re-used and obtained from public databases. Data citations in the article text are distinct from normal bibliographical citations and should directly link to the database records from which the data can be accessed. In the main text, data citations are formatted as follows: "Data ref: Smith et al, 2001" or "Data ref: NCBI Sequence Read Archive PRJNA342805, 2017". In the Reference list, data citations must be labeled with "[DATASET]". A data reference must provide the database name, accession number/identifiers and a resolvable link to the landing page from which the data can be accessed at the end of the reference. Further instructions are available at .

12) Please also note our reference format:
.

13) We now use CRediT to specify the contributions of each author in the journal submission system. CRediT replaces the author contribution section, which should be removed from the manuscript. Please use the free text box to provide more detailed descriptions. See also guide to authors:
.

14) As part of the EMBO publications' Transparent Editorial Process, EMBO reports publishes online a Review Process File to accompany accepted manuscripts. This File will be published in conjunction with your paper and will include the referee reports, your point-by-point response and all pertinent correspondence relating to the manuscript.

You can opt out of this by letting the editorial office know (emboreports@embo.org). If you do opt out, the Review Process File link will point to the following statement: "No Review Process File is available with this article, as the authors have chosen not to make the review process public in this case."

We would also welcome the submission of cover suggestions, or motifs to be used by our Graphics Illustrator in designing a cover.

I look forward to seeing a revised version of your manuscript when it is ready. Please let me know if you have any questions or comments regarding the revision.

You can use this link to submit your revision: https://embor.msubmit.net/cgi-bin/main.plex

Yours sincerely,

Ioannis Papaioannou, PhD
Editor
EMBO reports

Referee #1:

This manuscript by Cimadamore-Werthein, Baron et al is focused on kinetic analysis of the mitochondrion ADP/ATP carrier SLC25A4. Proposed transport mechanisms for this important protein have diverged significantly from earlier models invoking simultaneous binding of both substrates to a homodimeric transporter to more recent models based on a large body of structural and functional data, with seminal contributions from the Kunji group, revealing a monomeric transporter with a single binding site for ATP/ADP operating with an alternating access mechanism. While the latter is now widely accepted as being correct, experimental determination of transport kinetics has remained challenging. Here the authors use human SLC25A4 reconstituted in liposomes as the basis of a robotic transport assay that overcomes several challenges that have complicated accurate determination of reaction initial velocities and have led to error prone results and skewed interpretations of reaction kinetics. Assuming equivalent binding and transport steps for ATP and ADP, the assay measures uptake of radiolabeled ATP into proteoliposomes with different internal ATP concentrations. The authors are able to measure initial reaction rates based the linear time dependent accumulation of radiolabeled ATP inside liposomes. From these initial reaction rates at multiple external substrate concentrations, the authors are able to derive Michaelis Menten parameters for each internal ATP concentration. Plotting Km and Vmax values for each of these reveals parallel Lineweaver Burke plots, consistent with transport rates being independent of 'counter substrate' concentrations (internal ATP) and with a single binding site alternative access scheme. This represents an important and hitherto missing piece of functional evidence in further support of the so called 'ping-pong' kinetic model for transport that is likely applicable to other mitochondrial SLC25 family transporters. As such, the data presented are very much suitable for publication in EMBO reports as is. The manuscript is succinct and clearly written and was a pleasure to read. Parameterization of equations used for data fitting and their derivation are clearly explained. I only have minor points for clarification.

Minor Points:
1. Was there any reason only a single biological replicate is reported for the 2.5mM internal concentration of ATP?
2. Could the assay not be adapted for different concentrations of ADP inside by the addition of gramicidin or valinomycin allowing for Na+ or K+ influx into liposomes preloaded with Na+ inside and K+ outside or vice versa to mitigate the mentioned potential buildup with the ATP/ADP exchange?

Referee #2:

The mitochondrial ADP/ATP carrier (SLC25A4) is the prototype of proteins in the mitochondrial carrier family that transport nucleotides and various metabolites across the inner membrane. It is long believed to function as a homodimer in sequential mode in which the two exchanged substrates bind from the opposite sides of the membrane and move across the membrane simultaneously. The Kunji lab previously provided biochemical, biophysical and structural data that suggest a monomeric operation mode. In this model, there is only one single substrate binding site. An alternating access mechanism was proposed in which a central substrate binding site is used for substrate binding from one side of the membrane. Following conformational changes and the release of the substrate at the other side of the membrane, the transport counterion binds to the carrier to

complete the exchange process. However, this so-called ping-pong transport mechanism does not fit with previously published transport kinetic data, likely due to a number of confounding factors that might have deviated the final interpretation. In this manuscript, the authors reanalyzed the kinetic properties of human ADP/ATP carrier by using recombinant expression in yeast and reconstitution in proteoliposomes of defined lipid composition and buffer conditions. They captured time frames during the transport process in which the transport rate is linear. By measuring the uptake of radioactively labelled ATP, they found that the Km/Vmax ratio is constant when different internal concentrations of ATP are applied. The kinetic data support a ping-pong kinetic mechanism in which substrate exchange across the membrane occurs consecutively rather than simultaneously.
This is a well written short article with high-quality data that provide kinetic support for a monomeric operation mode of ADP/ATP carrier. The study is highly significant and has broad implications for the understanding of other mitochondrial carriers. I recommend the publication of the manuscript in EMBO report. I have only a few minor comments.

1. It would be helpful if the authors can explain why they choose to use a truncated version of Ant1.
2. I suggest moving Fig. 2A to Figure 1.
3. I also suggest that the authors include what is expected with regards to the Lineweaver-Burk lines and Km/Vmax values if the substrates are exchanged by a sequential mechanism. This may help those not in the field of enzymatic kinetics to better understanding the data.

Referee #3:

In this manuscript, the authors Cimadamore-Werthein, et. al studied kinetic properties of the human mitochondrial ADP/ATP carrier by using proteoliposomes and transport robotics. Based on the Km/Vmax ratio in varied internal ATP concentrations, the authors conclude that the carrier operates in a ping-pong mechanism rather than the early proposed sequential kinetic mechanism. In fact, the sequential kinetic mechanism on homodimer has not been mentioned for many years. In this proposed model, the c-state conformation always couples with m-state, so the ratio of the two states should always be 50%:50%. Therefore, the simplest and strongest evidence against this sequential mechanism is that the ADP/ATP carrier predominantly adopts the c-state or ADP-waiting conformation in rest condition (~98% based on previous NMR experiment, Nat Struct Mol Biol 2015, 22:636). Moreover, as early as 2007 (PNAS 104:10830), the same group of this manuscript reported that the carrier functions as monomer, which itself also excludes the possibility of sequential kinetic mechanism of homodimer. Therefore, the aim of this work seems not reasonable, especially when it's put within the context of the big publications of the authors.
The reviewer also wants to point out that although forming monomer excludes the possibility of sequential kinetic mechanism, the ping-pong mechanism does not exclude the possibility of forming dimmer. The ADP/ATP carrier functioning as monomer does not exclude the possibility that they still can form homodimer, especially when a large number of evidence suggests its existence (Biochem. Biophys. Res. Commun.1982,109:471; Biochemistry 1985,24:3821; Biochemistry 2016, 55:6238; etc). Forming homodimer does not mean that the movements of the two protomers have to couple with each other. Their association could be less specific, but still could be favorable mainly through the entropy contribution. Therefore, the reviewer think whether the ADP/ATP carrier exits in monomer or homodimer is still an issue of intensive controversy, and the author should not present it as a matter of fact. Moreover, the authors stated that the carrier has one single binding site, which is not supported by early biochemical results (Eur J Biochem 1970,16:313) and more recent results (Structure 2015, 23:1394; Comput Struct Biotechnol J 2022,20:1829).
Due to the above concerns, the current version of the manuscript is not acceptable, and the reviewer recommends the authors to interpret their results from a more reasonable angle and present their opinions in a more scientific and open-minded way when they resubmit their manuscript.

Dear Dr. Papaioannou,

We thank you and the reviewers for taking time to consider our manuscript. We are pleased that reviewers 1 and 2 appreciate the quality and significance of our work, but are surprised by the comments of reviewer 3. None of the latter deal with any aspect of the methods, experimental data or conclusions of this paper, but rather with secondary issues unrelated to the core message, which deals with the transport kinetics of the mitochondrial ADP/ATP carrier.

We have addressed the individual points below.

Referee #1:

This manuscript by Cimadamore-Werthein, Baron et al is focused on kinetic analysis of the mitochondrion ADP/ATP carrier SLC25A4. Proposed transport mechanisms for this important protein have diverged significantly from earlier models invoking simultaneous binding of both substrates to a homodimeric transporter to more recent models based on a large body of structural and functional data, with seminal contributions from the Kunji group, revealing a monomeric transporter with a single binding site for ATP/ADP operating with an alternating access mechanism. While the latter is now widely accepted as being correct, experimental determination of transport kinetics has remained challenging. Here the authors use human SLC25A4 reconstituted in liposomes as the basis of a robotic transport assay that overcomes several challenges that have complicated accurate determination of reaction initial velocities and have led to error prone results and skewed interpretations of reaction kinetics. Assuming equivalent binding and transport steps for ATP and ADP, the assay measures uptake of radiolabeled ATP into proteoliposomes with different internal ATP concentrations. The authors are able to measure initial reaction rates based the linear time dependent accumulation of radiolabeled ATP inside liposomes. From these initial reaction rates at multiple external substrate concentrations, the authors are able to derive Michaelis Menten parameters for each internal ATP concentration. Plotting Km and Vmax values for each of these reveals parallel Lineweaver Burke plots, consistent with transport rates being independent of 'counter substrate' concentrations (internal ATP) and with a single binding site alternative access scheme. This represents an important and hitherto missing piece of functional evidence in further support of the so called 'ping-pong' kinetic model for transport that is likely applicable to other mitochondrial SLC25 family transporters. As such, the data presented are very much suitable for publication in EMBO reports as is. The manuscript is succinct and clearly written and was a pleasure to read. Parameterization of equations used for data fitting and their derivation are clearly explained. I only have minor points for clarification.

We thank the reviewer for their detailed analysis of our manuscript and especially for the comment that this work is an 'important and hitherto missing piece of functional evidence in further support of the so called 'ping-pong' kinetic model for transport that is likely applicable to other mitochondrial SLC25 family transporters'.

Minor Points:

1. Was there any reason only a single biological replicate is reported for the 2.5mM internal concentration of ATP?

Our original internal ATP concentration series (0.1, 0.25, 0.5 and 1.0 mM) provided enough coverage to determine the kinetic mechanism of the carrier. To ensure our interpretation was correct at higher concentrations, we decided to test an internal ATP concentration of 2.5 mM, which is ~1000 times the Km of transport. As this single data set was consistent with the other data, we have included it for completeness, but have clearly stated that it represents one biological repeat, consisting of three technical repeats (still representing 21 individual data points).

2. Could the assay not be adapted for different concentrations of ADP inside by the addition of gramicidin or valinomycin allowing for Na+ or K+ influx into liposomes preloaded with Na+ inside and K+ outside or vice versa to mitigate the mentioned potential build up with the ATP/ADP exchange?

The reviewer is correct that these ionophores would prevent the build-up of a membrane potential, but they would also lead to changes in sodium or potassium concentrations, especially on the inside of the proteoliposomes, which have a very small volume. The activity of mitochondrial carriers is known to be sensitive to changes in ionic strength, because of the multiple ionic interactions involved in the mechanism, such as salt bridge network formation and substrate binding. Thus, to keep the experiment as simple as possible and to avoid any potential technical artefacts, we decided to use ATP homo-exchange, where the transport rates are solely dependent on the applied substrate gradients.

Referee #2:

The mitochondrial ADP/ATP carrier (SLC25A4) is the prototype of proteins in the mitochondrial carrier family that transport nucleotides and various metabolites across the inner membrane. It is long believed to function as a homodimer in sequential mode in which the two exchanged substrates bind from the opposite sides of the membrane and move across the membrane simultaneously. The Kunji lab previously provided biochemical, biophysical and structural data that suggest a monomeric operation mode. In this model, there is only one single substrate binding site. An alternating access mechanism was proposed in which a central substrate binding site is used for substrate binding from one side of the membrane. Following conformational changes and the release of the substrate at the other side of the membrane, the transport counterion binds to the carrier to complete the exchange process. However, this so-called ping-pong transport mechanism does not fit with previously published transport kinetic data, likely due to a number of confounding factors that might have deviated the final interpretation. In this manuscript, the authors reanalyzed the kinetic properties of human ADP/ATP carrier by using recombinant expression in yeast and reconstitution in proteoliposomes of defined lipid composition and buffer conditions. They captured time frames during the transport process in which the transport rate is linear. By measuring the uptake of radioactively labelled ATP, they found that the Km/Vmax ratio is

constant when different internal concentrations of ATP are applied. The kinetic data support a ping-pong kinetic mechanism in which substrate exchange across the membrane occurs consecutively rather than simultaneously.

This is a well written short article with high-quality data that provide kinetic support for a monomeric operation mode of ADP/ATP carrier. The study is highly significant and has broad implications for the understanding of other mitochondrial carriers. I recommend the publication of the manuscript in EMBO report. I have only a few minor comments.

We thank the reviewer for their supportive comments on the manuscript, kindly stating that this work is 'highly significant and has broad implications for the understanding of other mitochondrial carriers'.

1. It would be helpful if the authors can explain why they choose to use a truncated version of Ant1.

The full-length mammalian protein does not express in mitochondria of *S. cerevisiae* (Hatanaka et al., 2001; Jaiquel Baron et al., 2021). However, the truncation of the human carrier leads to high levels of expression and to complementation, and allows the purification of folded and functional protein (Jaiquel Baron et al., 2021). We have added a sentence to the manuscript to this effect.

2. I suggest moving Fig. 2A to Figure 1.

We thank the reviewer for their suggestion, and agree that it makes more sense to present Fig. 2A in Fig. 1, which we have now done.

3. I also suggest that the authors include what is expected with regards to the Lineweaver-Burk lines and Km/Vmax values if the substrates are exchanged by a sequential mechanism. This may help those not in the field of enzymatic kinetics to better understanding the data.

We thank the reviewer for their suggestion. We have added an additional supplementary figure to the manuscript, as suggested.

Referee #3:

In this manuscript, the authors Cimadamore-Werthein, et. al studied kinetic properties of the human mitochondrial ADP/ATP carrier by using proteoliposomes and transport robotics. Based on the Km/Vmax ratio in varied internal ATP concentrations, the authors conclude that the carrier operates in a ping-pong mechanism rather than the early proposed sequential kinetic mechanism. In fact, the sequential kinetic mechanism on homodimer has not been mentioned for many years. In this proposed model, the c-state conformation always couples with m-state, so the ratio of the two states should always be 50%:50%. Therefore, the simplest and strongest evidence against this sequential mechanism is that the ADP/ATP carrier predominantly adopts the c-state or ADP-waiting conformation in rest condition (~98%

based on previous NMR experiment, Nat Struct Mol Biol 2015, 22:636). Moreover, as early as 2007 (PNAS 104:10830), the same group of this manuscript reported that the carrier functions as monomer, which itself also excludes the possibility of sequential kinetic mechanism of homodimer. Therefore, the aim of this work seems not reasonable, especially when it's put within the context of the big publications of the authors.

We thank the reviewer for taking the time to read this paper, but we fundamentally disagree with their comments. Their remarks do not address any aspect of the methods, experimental data or conclusions of the paper, related to the kinetics of the mitochondrial ADP/ATP carrier, but rather deal with secondary issues. It is true that the kinetic mechanism has not been addressed for the longest time, but it was entirely proper to do so, as our results clearly contradict the earlier published data.

The reviewer also wants to point out that although forming monomer excludes the possibility of sequential kinetic mechanism, the ping-pong mechanism does not exclude the possibility of forming dimmer. The ADP/ATP carrier functioning as monomer does not exclude the possibility that they still can form homodimer, especially when a large number of evidence suggests its existence (Biochem. Biophys. Res. Commun.1982,109:471; Biochemistry 1985,24:3821; Biochemistry 2016, 55:6238; etc). Forming homodimer does not mean that the movements of the two protomers have to couple with each other. Their association could be less specific, but still could be favorable mainly through the entropy contribution. Therefore, the reviewer think whether the ADP/ATP carrier exits in monomer or homodimer is still an issue of intensive controversy, and the author should not present it as a matter of fact. Moreover, the authors stated that the carrier has one single binding site, which is not supported by early biochemical results (Eur J Biochem 1970,16:313) and more recent results (Structure 2015, 23:1394; Comput Struct Biotechnol J 2022,20:1829).

This work does not address whether the carrier operates as a monomer or dimer nor does it aim to identify the binding site of the carrier. It provides, in the words of one of the other reviewers, an ‘important and hitherto missing piece of functional evidence in further support of the so called 'ping-pong' kinetic model for transport’.

Due to the above concerns, the current version of the manuscript is not acceptable, and the reviewer recommends the authors to interpret their results from a more reasonable angle and present their opinions in a more scientific and open-minded way when they resubmit their manuscript.

A constant Km/Vmax ratio, which results in parallel lines in a Lineweaver-Burk plot, can only mean that substrate exchange follows a ping-pong kinetic mechanism. It is not a question of being reasonable. We are also not obliged to explain or justify the data of others in the publications the reviewer has mentioned, as none of them address the kinetic mechanism. However, in good scientific practice, we are allowed to present the most consistent mechanistic model that agrees with our kinetic data. It is somewhat ironic that we are accused of not being scientific or open-minded, since our results here overthrow a ~40-year-old dogma in the field.

We prefer to comment on the cited papers of the referee’s report below.

Biochem. Biophys. Res. Commun.1982,109:471 This paper does not deal with the kinetic mechanism of the ADP/ATP carrier, but investigates the oligomeric state by neutron scattering, claiming it was a dimer. At the time, the protein was purified by negative chromatography, meaning that large amounts of lipids and detergent co-purified, which could not be fully masked by deuterium, leading to an overestimation of the size, as found later (Nury et al., 2008).

Eur J Biochem 1970, 16:313 This paper does not deal with the kinetic mechanism, but instead studies the competition between atractyloside and substrates, showing that they compete for the same site, which has been supported by the structures (Pebay-Peyroula et al., 2003; Ruprecht et al., 2014).

Nat Struct Mol Biol 2015, 22:636 In this NMR study the claim is made that AAC is "refolded" from inclusion bodies in Fos-choline-12, which is one of the harshest detergents. Under these conditions, the specific inhibitor carboxyatractyloside has a Kd of 20 uM, which is at least four orders of magnitude higher than that of the native protein, demonstrating that the protein is misfolded, Thus, none of the claims in this paper are valid, as all subsequent work has also shown. We and others have already addressed these and other issues with this work extensively (Chipot et al., 2018; Dehez et al., 2017; King et al., 2018; Kurauskas et al., 2018a; Kurauskas et al., 2018b).

PNAS 2007, 104:10830 This paper does not address the kinetic mechanism, but demonstrates that the mitochondrial ADP/ATP carrier functions as a monomer in mitochondrial membranes, which is consistent with our proposed structural mechanism (Figure 3).

Biochemistry 2016, 55:6238 This paper does not address the kinetic or molecular mechanism of the carrier. In this paper coarse grain dynamics are performed on a set of inactive carriers to show that they clump together to form dimers, trimers, tetramers, and hexamers, possibly because ionic strength and pH are difficult to model well in coarse grain simulations. Importantly, they show that there is no conserved dimerization interface, as carriers interact with all three domains randomly. Still, none of these conclusions are relevant, as in reality the carriers cycle continuously between a cytoplasmic and matrix conformation, each carrier being in a different dynamic state at any one time, precluding stable interactions. We have addressed these and other issues with this study previously (Kunji and Ruprecht, 2020).

Biochemistry 1985, 24:3821 This paper shows that the carrier from beef heart mitochondria has three tightly bound cardiolipins per protein, as revealed by ^{31}P NMR. This claim has indeed been confirmed by structural studies (Nury et al., 2005; Pebay-Peyroula et al., 2003; Ruprecht et al., 2014; Ruprecht et al., 2019), but it does not address the kinetic mechanism or oligomeric state of the protein.

Structure 2015, 23:1394 This is a study with a completely different carrier, the Mg-ATP/Pi carrier, and does not address any aspects of our paper. Again, this work used fos-choline-12 and thus has no relevant functional information, because the protein was misfolded (see comments and papers above).

Comput Struct Biotechnol J 2022, 20:1829 This is a molecular dynamics paper that does not address the kinetic mechanism. However, it does claim that the carrier in the cytoplasmic state has a second binding site, involving the positively charged residues of the cytoplasmic network. However, the cytoplasmic network is universal to all SLC25 carriers, thus providing no molecular basis for substrate specificity, but it does have a defined and experimentally proven role in the mechanism (King et al., 2016; Ruprecht et al., 2019). The suggested second binding site is located in the region that forms the cytoplasmic gate, meaning that substrate binding and closure of the carrier are mutually exclusive.

In fact, MD simulations have identified hundreds of potential binding sites and substrate poses, but none of these studies are carried out in a relevant time frame to assess their significance for the transport mechanism, which occurs on millisecond time scale rather than microsecond time scale. Moreover, we have not been able to confirm the involvement of these additional sites in substrate binding using thermostability shift assays, which only detect significant substrate-carrier interactions (Mavridou et al., 2022). Thus, the ADP/ATP carrier has only one substrate binding site in agreement with a ping-pong kinetic mechanism, demonstrated unequivocally by this work.

References

Chipot, C., Dehez, F., Schnell, J.R., Zitzmann, N., Pebay-Peyroula, E., Catoire, L.J., Miroux, B., Kunji, E.R.S., Veglia, G., Cross, T.A.*, et al.* (2018). Perturbations of native membrane protein structure in alkyl phosphocholine detergents: a critical assessment of NMR and biophysical studies. Chem Rev *118*, 3559-3607.

Dehez, F., Schanda, P., King, M.S., Kunji, E.R.S., and Chipot, C. (2017). Mitochondrial ADP/ATP carrier in dodecylphosphocholine binds cardiolipins with non-native affinity. Biophys J *113*, 2311-2315.

Hatanaka, T., Hashimoto, M., Majima, E., Shinohara, Y., and Terada, H. (2001). Significant effect of the N-terminal region of the mitochondrial ADP/ATP carrier on its efficient expression in yeast mitochondria. J Biol Chem *276*, 28881-28888.

Jaiquel Baron, S., King, M.S., Kunji, E.R.S., and Schirris, T.J.J. (2021). Characterization of drug-induced human mitochondrial ADP/ATP carrier inhibition. Theranostics *11*, 5077-5091.

King, M.S., Crichton, P.G., Ruprecht, J.J., and Kunji, E.R.S. (2018). Concerns with yeast mitochondrial ADP/ATP carrier's integrity in DPC. Nat Struct Mol Biol *25*, 747-749.

King, M.S., Kerr, M., Crichton, P.G., Springett, R., and Kunji, E.R.S. (2016). Formation of a cytoplasmic salt bridge network in the matrix state is a fundamental step in the transport mechanism of the mitochondrial ADP/ATP carrier. Biochim Biophys Acta *1857*, 14-22.

Kunji, E.R.S., and Ruprecht, J.J. (2020). The mitochondrial ADP/ATP carrier exists and functions as a monomer. Biochem Soc Trans.

Kurauskas, V., Hessel, A., Dehez, F., Chipot, C., Bersch, B., and Schanda, P. (2018a). Dynamics and interactions of AAC3 in DPC are not functionally relevant. Nat Struct Mol Biol *25*, 745-747.

Kurauskas, V., Hessel, A., Ma, P., Lunetti, P., Weinhaupl, K., Imbert, L., Brutscher, B., King, M.S., Sounier, R., Dolce, V.*, et al.* (2018b). How Detergent Impacts Membrane Proteins: Atomic-Level Views of Mitochondrial Carriers in Dodecylphosphocholine. J Phys Chem Lett *9*, 933-938.

Mavridou, V., King, M.S., Tavoulari, S., Ruprecht, J.J., Palmer, S.M., and Kunji, E.R.S. (2022). Substrate binding in the mitochondrial ADP/ATP carrier is a step-wise process guiding the structural changes in the transport cycle. Nat Commun *13*, 3585.

Nury, H., Dahout-Gonzalez, C., Trezeguet, V., Lauquin, G., Brandolin, G., and Pebay-Peyroula, E. (2005). Structural basis for lipid-mediated interactions between mitochondrial ADP/ATP carrier monomers. FEBS Lett *579*, 6031-6036.

Nury, H., Manon, F., Arnou, B., le Maire, M., Pebay-Peyroula, E., and Ebel, C. (2008). Mitochondrial bovine ADP/ATP carrier in detergent is predominantly monomeric, but also forms multimeric species. Biochemistry *43*, 12319-12331.

Pebay-Peyroula, E., Dahout-Gonzalez, C., Kahn, R., Trezeguet, V., Lauquin, G.J., and Brandolin, G. (2003). Structure of mitochondrial ADP/ATP carrier in complex with carboxyatractyloside. Nature *426*, 39-44.

Ruprecht, J.J., Hellawell, A.M., Harding, M., Crichton, P.G., McCoy, A.J., and Kunji, E.R. (2014). Structures of yeast mitochondrial ADP/ATP carriers support a domain-based alternating-access transport mechanism. Proc Natl Acad Sci U S A *111*, E426-434.

Ruprecht, J.J., King, M.S., Zogg, T., Aleksandrova, A.A., Pardon, E., Crichton, P.G., Steyaert, J., and Kunji, E.R.S. (2019). The molecular mechanism of transport by the mitochondrial ADP/ATP carrier. Cell *176*, 435–447.

1st Revision - Editorial Decision 11th May 2023

Dear Edmund,

Thank you for submitting your revised manuscript for consideration by EMBO reports. It has now been seen by the three referees who were asked to re-evaluate your manuscript, and we have received the full set of their reports that I have already shared with you (included again below), as well as cross-referee comments from them. Referees #1 and #2 mention that their concerns have been addressed, and they now support publication of the paper in EMBO reports, while referee #3 suggests that a clarification of the data interpretation be added to the manuscript.

I would also like to thank you for your feedback on the outcome of the above process and your willingness to include a statement in the Discussion of your manuscript clarifying that the data presented in this paper do not directly address the question of the oligomeric state of the ADP/ATP carrier, in response to the criticism of referee #3. Please include your suggested text in a revised manuscript, and make sure that the changes are highlighted (or "tracked") to be clearly visible. Please also include in your re-submission a response/rebuttal note to the comments of referee #3.

From the editorial side, there are also a few things that we need from you before we can proceed with acceptance of your manuscript:

- You can either publish the study as a Report or as an Article. For Reports, the revised manuscript should not exceed 27,000 characters (including spaces but excluding Materials & Methods and references) and 5 main plus 5 Expanded View figures. The Results and Discussion sections must be combined, which will help to shorten the manuscript text by eliminating some redundancy that is inevitable when discussing the same experiments twice. For an Article there are no length limitations, but it should have more than 5 main figures and the Results and Discussion sections must be separate. In both cases, the entire Materials and Methods must be included in the main manuscript file.

- Please provide up to 5 keywords in your revised manuscript (you currently have 6).

- Please update your data availability statement: since your study does not include any datasets requiring deposition in a public database, the statement should be: "This study includes no data deposited in external repositories." under the heading "Data availability" at the end of Materials and Methods.

- The author contributions statement should be removed from the manuscript file. Instead, we now use CRediT to specify the contributions of each author in the journal submission system. Please use the free text box to provide more detailed descriptions. See also our guide to authors: <https://www.embopress.org/page/journal/14693178/authorguide#authorshipguidelines>.

- Please upload your synopsis image as a separate image file (pixel dimensions: 550 x 300-600 -width x height-, preferably in .jpg or .png format).

- We noticed that the source data checklist (that our source data coordinator, Dr. Hannah Sonntag, has previously sent you) is missing: please include a completed source data checklist in your re-submission. Please contact Dr. Sonntag (h.sonntag@source-data.org) directly if you have any questions regarding source data.

- Your manuscript has been inspected by our data editors for completeness and accuracy. Please see the required changes in the attached Word file and address all comments in your revised manuscript.

Please also note that as part of the EMBO publications' Transparent Editorial Process, EMBO reports publishes online a Review Process File to accompany accepted manuscripts. This File will be published in conjunction with your paper and will include the referee reports, your point-by-point response and all pertinent correspondence relating to the manuscript.

You can opt out of this by letting the editorial office know (emboreports@embo.org). If you do opt out, the Review Process File link will point to the following statement: "No Review Process File is available with this article, as the authors have chosen not to make the review process public in this case."

We would also welcome the submission of cover suggestions or motifs to be used by our Graphics Illustrator in designing a cover.

We look forward to seeing a final version of your manuscript as soon as possible. Please use this link to submit your revision: https://embor.msubmit.net/cgi-bin/main.plex

Best regards,

Ioannis

Ioannis Papaioannou, PhD
Editor
EMBO reports

Referee #1:

The authors have adequately addressed the minor points I had, and I look forward to seeing this manuscript out in its final form.

Referee #2:

The reviewer has no further comments.

Referee #3:

The authors didn't address most of my concerns. As I stated what I really conerned are not the methods and results of this work but the proposed transport mechanism framework the authors used to understand these results. The authors proposed a symmetric transport mechanism, based on which they insisted that the carrier can not exist as dimeric form, and that's the reason why thet symmetrized the asymmetric m-state structure in their previous Cell paper. However, I couldn't find the basis for their proposal for a symmetric transport mechanism. In fact there are a variety of evidences suggesting an asymmetric transport mechanism. Therefore, the authors' proposal could quite possibly be wrong, or at least the authors should soften their statement as possibly as they can in their manuscirpt.

Referee #1

The authors have adequately addressed the minor points I had, and I look forward to seeing this manuscript out in its final form.

Thank you for your helpful comments to improve our manuscript.

Referee #2

The reviewer has no further comments.

Thank you for your helpful comments to improve our manuscript.

Referee #3

The authors didn't address most of my concerns. As I stated what I really conerned are not the methods and results of this work but the proposed transport mechanism framework the authors used to understand these results. The authors proposed a symmetric transport mechanism, based on which they insisted that the carrier can not exist as dimeric form, and that's the reason why thet symmetrized the asymmetric m-state structure in their previous Cell paper. However, I couldn't find the basis for their proposal for a symmetric transport mechanism. In fact there are a variety of evidences suggesting an asymmetric transport mechanism. Therefore, the authors' proposal could quite possibly be wrong, or at least the authors should soften their statement as possibly as they can in their manuscirpt.

In this paper, we do not address whether the mechanism is symmetric or asymmetric.

To comply with the reviewer's request, we have added the following line to the discussion:

The data presented in this paper do not directly address the question of the oligomeric state of the ADP/ATP carrier. However, they provide further support for the notion that the carrier functions as a monomer with a single substrate binding site, consistent with a large number of structural and functional data gathered over recent years.

2nd Revision - Editorial Decision 16th May 2023

Dear Edmund,

Thank you for the submission of your revised manuscript to our offices. Browsing through the manuscript, we have noticed that there are still two issues that need to be fixed before we can proceed with acceptance of your paper. In particular:

1. Please note that you can either publish the study as a Report or as an Article. For Reports, the revised manuscript should not exceed 27,000 characters (including spaces but excluding Materials & Methods and references) and 5 main plus 5 Expanded View figures. The Results and Discussion sections must be combined, which will help to shorten the manuscript text by eliminating some redundancy that is inevitable when discussing the same experiments twice. For an Article there are no length limitations, but it should have more than 5 main figures and the Results and Discussion sections must be separate. In both cases, the entire Materials and Methods must be included in the main manuscript file.

2. The author contributions statement should be removed from the manuscript file. Instead, we use CRediT to specify the contributions of each author in the journal submission system. Please use the free text box to provide more detailed descriptions. See also our guide to authors:
<https://www.embopress.org/page/journal/14693178/authorguide#authorshipguidelines>.

Please address both issues in a revised manuscript. I look forward to seeing the new version as soon as possible. Please use this link to submit your revision: https://embor.msubmit.net/cgi-bin/main.plex

Best regards,

Ioannis

Ioannis Papaioannou, PhD
Editor
EMBO reports

All editorial and formatting issues were resolved by the authors.

3rd Revision - Editorial Decision 17th May 2023

Prof. Edmund Kunji
University of Cambridge
MRC Mitochondrial Biology Unit
Wellcome Trust/MRC building
Hills road
Cambridge, Cambridgeshire CB2 0XY
United Kingdom

Dear Edmund,

I am very pleased to accept your manuscript for publication in the next available issue of EMBO reports. Thank you for your contribution to our journal.

At the end of this email I include important information about how to proceed. Please ensure that you take the time to read the information and complete and return the necessary forms to allow us to publish your manuscript as quickly as possible.

As part of the EMBO publication's Transparent Editorial Process, EMBO reports publishes online a Review Process File to accompany accepted manuscripts. As you are aware, this File will be published in conjunction with your paper and will include the referee reports, your point-by-point response and all pertinent correspondence relating to the manuscript.

If you do NOT want this File to be published, please inform the editorial office within 2 days, if you have not done so already, otherwise the File will be published by default [contact: emboreports@embo.org]. If you do opt out, the Review Process File link will point to the following statement: "No Review Process File is available with this article, as the authors have chosen not to make the review process public in this case."

Should you be planning a Press Release on your article, please get in contact with emboreports@wiley.com as early as possible, in order to coordinate publication and release dates.

Thank you again for your contribution to EMBO reports and congratulations on a successful publication. Please consider us again in the future for your most exciting work.

Best regards,

Ioannis

Ioannis Papaioannou, PhD
Editor
EMBO reports

****

THINGS TO DO NOW:

Please note that you will be contacted by Wiley Author Services to complete licensing and payment information. The required 'Page Charges Authorization Form' is available here: https://www.embopress.org/pb-assets/embo-site/er_apc.pdf - please download and complete the form and return to embopressproduction@wiley.com

EMBO Press participates in many Publish and Read agreements that allow authors to publish Open Access with reduced/no publication charges. Check your eligibility: https://authorservices.wiley.com/author-resources/Journal-Authors/open-access/affiliation-policies-payments/index.html

You will receive proofs by e-mail approximately 2-3 weeks after all relevant files have been sent to our Production Office; you should return your corrections within 2 days of receiving the proofs.

Please inform us if there is likely to be any difficulty in reaching you at the above address at that time. Failure to meet our deadlines may result in a delay of publication, or publication without your corrections.

All further communications concerning your paper should quote reference number EMBOR-2023-57127V4 and be addressed to

emboreports@wiley.com.

Should you be planning a Press Release on your article, please get in contact with emboreports@wiley.com as early as possible, in order to coordinate publication and release dates.

EMBO Press Author Checklist

Corresponding Author Name: EDMUND KUNJI	
Journal Submitted to: EMBO REPORTS	
Manuscript Number: EMBOR-2023-57127	

USEFUL LINKS FOR COMPLETING THIS FORM
The EMBO Journal - Author Guidelines
EMBO Reports - Author Guidelines
Molecular Systems Biology - Author Guidelines
EMBO Molecular Medicine - Author Guidelines

Reporting Checklist for Life Science Articles (updated January
This checklist is adapted from Materials Design Analysis Reporting (MDAR) Checklist for Authors. MDAR establishes a minimum set of requirements in transparent reporting in the life sciences (see Statement of Task: 10.31222/osf.io/9sm4x). Please follow the journal's guidelines in preparing your
Please note that a copy of this checklist will be published alongside your article.

Abridged guidelines for figures
1. Data
The data shown in figures should satisfy the following conditions:
- the data were obtained and processed according to the field's best practice and are presented to reflect the results of the experiments in an accurate and unbiased manner.
- ideally, figure panels should include only measurements that are directly comparable to each other and obtained with the same assay.
- plots include clearly labeled error bars for independent experiments and sample sizes. Unless justified, error bars should not be shown for technical
- if n<5, the individual data points from each experiment should be plotted. Any statistical test employed should be justified.
- Source Data should be included to report the data underlying figures according to the guidelines set out in the authorship guidelines on Data

2. Captions
Each figure caption should contain the following information, for each panel where they are relevant:
- a specification of the experimental system investigated (eg cell line, species name).
- the assay(s) and method(s) used to carry out the reported observations and measurements.
- an explicit mention of the biological and chemical entity(ies) that are being measured.
- an explicit mention of the biological and chemical entity(ies) that are altered/varied/perturbed in a controlled manner.
- the exact sample size (n) for each experimental group/condition, given as a number, not a range;
- a description of the sample collection allowing the reader to understand whether the samples represent technical or biological replicates (including how many animals, litters, cultures, etc.).
- a statement of how many times the experiment shown was independently replicated in the laboratory.
- definitions of statistical methods and measures:
  - common tests, such as t-test (please specify whether paired vs. unpaired), simple χ2 tests, Wilcoxon and Mann-Whitney tests, can be unambiguously identified by name only, but more complex techniques should be described in the methods section;
  - are tests one-sided or two-sided?
  - are there adjustments for multiple comparisons?
  - exact statistical test results, e.g., P values = x but not P values < x;
  - definition of 'center values' as median or average;
  - definition of error bars as s.d. or s.e.m.

Please complete ALL of the questions below.
Select "Not Applicable" only when the requested information is not relevant for your study.

Materials

Newly Created Materials	Information included in the manuscript?	In which section is the information available? (Reagents and Tools Table, Materials and Methods, Figures, Data Availability Section)
New materials and reagents need to be available; do any restrictions apply?	Not Applicable	

Antibodies	Information included in the manuscript?	In which section is the information available? (Reagents and Tools Table, Materials and Methods, Figures, Data Availability Section)
For **antibodies** provide the following information:<br>- Commercial antibodies: RRID (if possible) or supplier name, catalogue number and or/clone number<br>- Non-commercial: RRID or citation	Not Applicable	

DNA and RNA sequences	Information included in the manuscript?	In which section is the information available? (Reagents and Tools Table, Materials and Methods, Figures, Data Availability Section)
**Short novel DNA or RNA including primers, probes**: provide the sequences.	Not Applicable	

Cell materials	Information included in the manuscript?	In which section is the information available? (Reagents and Tools Table, Materials and Methods, Figures, Data Availability Section)
**Cell lines:** Provide species information, strain. Provide accession number in repository **OR** supplier name, catalog number, clone number, and/**OR** RRID.	Not Applicable	
**Primary cultures:** Provide species, strain, sex of origin, genetic modification status.	Not Applicable	
Report if the cell lines were recently **authenticated** (e.g., by STR profiling) and tested for mycoplasma contamination.	Not Applicable	

Experimental animals	Information included in the manuscript?	In which section is the information available? (Reagents and Tools Table, Materials and Methods, Figures, Data Availability Section)
**Laboratory animals or Model organisms:** Provide species, strain, sex, age, genetic modification status. Provide accession number in repository **OR** supplier name, catalog number, clone number, **OR** RRID.	Not Applicable	
**Animal observed in or captured from the field:** Provide species, sex, and age where possible.	Not Applicable	
Please detail **housing and husbandry conditions**.	Not Applicable	

Plants and microbes	Information included in the manuscript?	In which section is the information available? (Reagents and Tools Table, Materials and Methods, Figures, Data Availability Section)
**Plants:** provide species and strain, ecotype and cultivar where relevant, unique accession number if available, and source (including location for collected wild specimens).	Not Applicable	
**Microbes:** provide species and strain, unique accession number if available, and source.	Yes	MATERIALS AND METHODS

Human research participants	Information included in the manuscript?	In which section is the information available? (Reagents and Tools Table, Materials and Methods, Figures, Data Availability Section)
If collected and within the bounds of privacy constraints report on age, sex and gender or ethnicity for all study participants.	Not Applicable	

Core facilities	Information included in the manuscript?	In which section is the information available? (Reagents and Tools Table, Materials and Methods, Figures, Data Availability Section)
If your work benefited from core facilities, was their service mentioned in the acknowledgments section?	Yes	ACKNOWLEDGEMENTS

Design

Study protocol	Information included in the manuscript?	In which section is the information available? (Reagents and Tools Table, Materials and Methods, Figures, Data Availability Section)
If study protocol has been **pre-registered, provide DOI in the manuscript**. For clinical trials, provide the trial registration number **OR** cite DOI.	Not Applicable	
Report the **clinical trial registration number** (at ClinicalTrials.gov or equivalent), where applicable.	Not Applicable	

Laboratory protocol	Information included in the manuscript?	In which section is the information available? (Reagents and Tools Table, Materials and Methods, Figures, Data Availability Section)
Provide DOI OR other citation details if **external detailed step-by-step protocols** are available.	Yes	MATERIALS AND METHODS

Experimental study design and statistics	Information included in the manuscript?	In which section is the information available? (Reagents and Tools Table, Materials and Methods, Figures, Data Availability Section)
Include a statement about **sample size** estimate even if no statistical methods were used.	Yes	FIGURES
Were any steps taken to minimize the effects of subjective bias when allocating animals/samples to treatment (e.g. **randomization procedure**)? If yes, have they been described?	Not Applicable	
Include a statement about **blinding** even if no blinding was done.	Not Applicable	
Describe **inclusion/exclusion criteria** if samples or animals were excluded from the analysis. Were the criteria pre-established?<br><br>If sample or data points were omitted from analysis, report if this was due to attrition or intentional exclusion and provide justification.	Not Applicable	
For every figure, are **statistical tests** justified as appropriate? Do the data meet the assumptions of the tests (e.g., normal distribution)? Describe any methods used to assess it. Is there an estimate of variation within each group of data? Is the variance similar between the groups that are being statistically compared?	Not Applicable	

Sample definition and in-laboratory replication	Information included in the manuscript?	In which section is the information available? (Reagents and Tools Table, Materials and Methods, Figures, Data Availability Section)
In the figure legends: state number of times the experiment was **replicated** in laboratory.	Yes	FIGURES
In the figure legends: define whether data describe **technical or biological replicates**.	Yes	FIGURES

Ethics

Ethics	Information included in the manuscript?	In which section is the information available? (Reagents and Tools Table, Materials and Methods, Figures, Data Availability Section)
Studies involving **human participants**: State details of **authority granting ethics approval** (IRB or equivalent committee(s), provide reference number for approval.	Not Applicable	
Studies involving **human participants**: Include a statement confirming that **informed consent** was obtained from all subjects and that the experiments conformed to the principles set out in the WMA Declaration of Helsinki and the Department of Health and Human Services Belmont Report.	Not Applicable	
Studies involving **human participants:** For publication of **patient photos**, include a statement confirming that consent to publish was obtained.	Not Applicable	
Studies involving experimental **animals**: State details of **authority granting ethics approval** (IRB or equivalent committee(s), provide reference number for approval. Include a statement of compliance with ethical regulations.	Not Applicable	
Studies involving **specimen and field samples:** State if relevant **permits** obtained, provide details of authority approving study; if none were required, explain why.	Not Applicable	

Dual Use Research of Concern (DURC)	Information included in the manuscript?	In which section is the information available? (Reagents and Tools Table, Materials and Methods, Figures, Data Availability Section)
Could your study fall under dual use research restrictions? Please check biosecurity documents and list of **select agents and toxins** (CDC): https://www.selectagents.gov/sat/list.htm	Not Applicable	
If you used a select agent, is the security level of the lab appropriate and reported in the manuscript?	Not Applicable	
If a study is subject to dual use research of concern regulations, is the name of the **authority granting approval and reference number** for the regulatory approval provided in the manuscript?	Not Applicable	

Reporting

The MDAR framework recommends adoption of discipline-specific guidelines, established and endorsed through community initiatives. Journals have their own policy about requiring specific guidelines and recommendations to complement MDAR.

Adherence to community standards	Information included in the manuscript?	In which section is the information available? (Reagents and Tools Table, Materials and Methods, Figures, Data Availability Section)
State if relevant guidelines or checklists (e.g., **ICMJE, MIBBI, ARRIVE, PRISMA**) have been followed or provided.	Not Applicable	
For **tumor marker prognostic studies,** we recommend that you follow the **REMARK** reporting guidelines (see link list at top right). See author guidelines, under 'Reporting Guidelines'. Please confirm you have followed these guidelines.	Not Applicable	
For **phase II and III randomized controlled trials**, please refer to the **CONSORT** flow diagram (see link list at top right) and submit the CONSORT checklist (see link list at top right) with your submission. See author guidelines, under 'Reporting Guidelines'. Please confirm you have submitted this list.	Not Applicable	

Data Availability

Data availability	Information included in the manuscript?	In which section is the information available? (Reagents and Tools Table, Materials and Methods, Figures, Data Availability Section)
Have **primary datasets** been deposited according to the journal's guidelines (see 'Data Deposition' section) and the respective accession numbers provided in the Data Availability Section?	Not Applicable	
Were **human clinical and genomic datasets** deposited in a public access-controlled repository in accordance to ethical obligations to the patients and to the applicable consent agreement?	Not Applicable	
Are **computational models** that are central and integral to a study available without restrictions in a machine-readable form? Were the relevant accession numbers or links provided?	Not Applicable	
If publicly available data were reused, provide the respective **data citations in the reference list**.	Not Applicable	
